# A Fiber-Coupled Self-Mixing Laser Diode for the Measurement of Young’s Modulus

**DOI:** 10.3390/s16060928

**Published:** 2016-06-22

**Authors:** Ke Lin, Yanguang Yu, Jiangtao Xi, Huijun Li, Qinghua Guo, Jun Tong, Lihong Su

**Affiliations:** 1 School of Electrical, Computer and Telecommunications Engineering, University of Wollongong, Northfields Ave, Wollongong NSW 2522, Australia; kl740@uowmail.edu.au (K.L.); jiangtao@uow.edu.au (J.X.); qguo@uow.edu.au (Q.G.); jtong@uow.edu.au (J.T.); 2 School of Mechanical, Materials and Mechatronic Engineering, University of Wollongong, Wollongong NSW 2522, Australia; huijun@uow.edu.au (H.L.); lihongsu@uow.edu.au (L.S.)

**Keywords:** fiber-coupled, self-mixing laser diode, self-mixing interferometry, fundamental resonant frequency, Young’s modulus

## Abstract

This paper presents the design of a fiber-coupled self-mixing laser diode (SMLD) for non-contact and non-destructive measurement of Young’s modulus. By the presented measuring system, the Young’s modulus of aluminum 6061 and brass are measured as 70.0 GPa and 116.7 GPa, respectively, showing a good agreement within the standards in the literature and yielding a much smaller deviation and a higher repeatability compared with traditional tensile testing. Its fiber-coupled characteristics make the system quite easy to be installed in many application cases.

## 1. Introduction

Young’s modulus is defined as the ratio of stress to strain during the elastic loading, which plays a vital role for investigation of the stability and reliability of devices and to evaluate the performance and longevity under certain pressure or tension. Conventional methods for modulus measurement are more based on tensile test [1], three-point bending test or indentation [2]. However, these methods usually need a dedicated test setup and might not be feasible to carry out in a time and cost effective way. In addition, it is difficult to inspect the changes of modulus on a test specimen during storage under stress conditions as further degradation progresses the specimen would risk an irreversible deformation.

Resonant methods recently have attracted a large amount of researchers for measurement of Young’s modulus and material related property. As Young’s modulus influences the vibration behavior of material structures, the vibration behavior of a specific specimen can provide the materials’ modulus. Impulse excitation method is one kind of these techniques, which are based on measurement of resonant frequency in terms of longitudinal or flexural vibration of the test specimen with simple geometry (basically a circular plate, a cylinder or a prism with uniform rectangular cross-section) [3,4,5,6]. The test specimen can be impacted to vibrate at the resonant frequency by a singular mechanical strike [4] or by a driver that persistently varies the frequency of the output signal [5], or even in a photothermic or acoustic way [3,6]. Comparing with the traditional modulus measuring methods, which are often destructive and cost consuming, impulse excitation approach presents its superiority, because of its ease of specimen preparation, a variety of test specimen shapes, high accuracy, and even measurement in a hostile environment [7]. It has been extensively used for measurement of various kinds of materials [5,6,7,8,9,10], even for human or animal organs [11].

Recently, optical techniques have been attractive for measuring mechanical properties, such as the application of the laser sensor [12,13,14], the interference of light beams [15], atomic force microscope [16], and electronic speckle pattern interferometry [17] and so on. Optical feedback self-mixing interferometry (SMI) technique, a new kind of laser interferometry, is an effective way to measure the vibration period and displacement of the external target, even some important useful material parameters. When the laser emitted by the laser diode (LD) is reflected or backscattered from the external target and re-enter into the laser cavity again, it will mix and interfere with the original laser, thus generating a modulated signal, whose frequency and amplitude will change [18]. Thus, based on SMI signal, the system can be used to retrieve the useful information about the external target, such as Young’s modulus. Unlike most optical methods that separate the laser source and interferometer to split and combine the beam, self-mixing is based on the interaction between cavity field and the one backscattered from the target. Because of its simplicity, convenience, feasibility of operation on many diffusive surfaces and the high sensitivity of the scheme, being a sort of coherent detection that easily attains half-wavelength resolution, even a few tens nm resolution [19], the SMI technique is considered an effective solution for non-contact measurement of vibration and displacement [20].

Previously, we have done preliminary work on feasibility of using self-mixing laser diode (SMLD) for measuring Young’s modulus, including basic experimental system set-up and signal processing method [21,22]. In this paper, we introduced fiber to the system, which makes the installation of measuring system more flexible. The details on the overall system design and signal analysis method are presented. Section 2 gives the principle in terms of the formula used, the generation and the acquisition of the vibrating signal. Then, we elaborate the design procedures of the measuring system in Section 3 regarding to the support needed for the specimen and the size of the impulse tool that is used to excite the specimen, as well as the optical requirements for fiber-coupled SMLD system. Simulations and experiments are performed in Section 4 and Section 5, respectively. An experimental comparison is also conducted between current SMI technique and the traditional tensile testing. Section 6 concludes the paper.

## 2. Measurement Principle

### 2.1. Measurement Formula for Young’s Modulus

**Figure 1:**
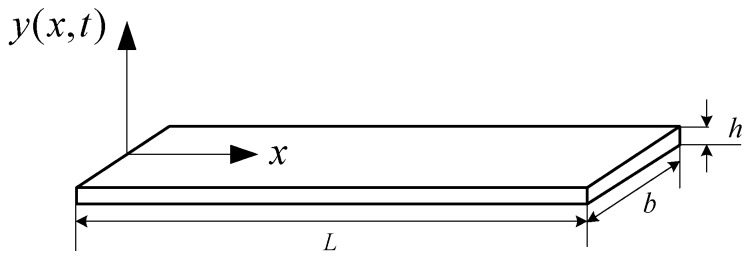
Specimen and its vibrating coordinate system.

Young’s modulus (denoted by E) can be calculated based on the geometry dimension of a specimen and its fundamental resonant frequency (denoted by $f_{RO}$) [4]. A rectangular specimen (L: length, b: width, h: thickness) is shown in Figure 1. According to the standard released by ASTM E187621, the calculation formula of E is expressed as below while L/h≥20:

(1) $$E=0.9465\cdot \frac{mf_{RO}{}^{2}L^{3}}{bh^{3}}\cdot T$$

where

(2) $$T=1+6.585{\left(h/L\right)}^{2}$$

m is the mass of the specimen and T is the correction factor.

### 2.2. Vibrating Signals Generated by the Test Specimen

The fundamental resonant frequency in Equation (1) is carried in a vibrating signal generated by the test specimen. A rectangular specimen can be stimulated and vibrated at its fundamental vibration mode. If setting the coordinate system shown in Figure 1, the vibration waveform y(x,t) at any point along x-axis varying with time t can be described by the following differential equation [23].

(3) $$EI\frac{\partial^{4}y(x,t)}{\partial x^{4}}+\rho A\frac{\partial^{2}y(x,t)}{\partial t^{2}}+\gamma \frac{\partial y(x,t)}{\partial t}=0\;\left(0\leq x\leq L\right)$$

where $\gamma \frac{\partial y(x,t)}{\partial t}$ models for internal energy loss mechanism of the specimen, and I, ρ and A, respectively, represent for the area moment of inertia, the density and the cross section area of the specimen. After separating the variable of x and t, and solving Equation (3), we can express y(x,t) as below:

(4) $$y(x,t)=y_{n}(x)\cdot e^{-\zeta \omega_{n}t}\cos(\omega_{n}\sqrt{1-\zeta^{2}}t+\varphi )\;(n=1,2,3...)$$

where

(5) $$y_{n}(x)=-A_{0}\left\{\begin{array}{l} \cosh\left((\beta_{n}L)\frac{x}{L}\right)+\cos\left((\beta_{n}L)\frac{x}{L}\right)\\ -\frac{\cosh(\beta_{n}L)-\cos(\beta_{n}L)}{\sinh(\beta_{n}L)-\sin(\beta_{n}L)}\left[\sinh\left((\beta_{n}L)\frac{x}{L}\right)+\sin\left((\beta_{n}L)\frac{x}{L}\right)\right] \end{array}\right\}$$

In Equation (4), n stands for order of the vibration mode; ζ is the damping ratio (typically ζ=0.001~0.002); $\omega_{n}(=2\pi f_{n})$ describes the natural angular frequency of the *n*th order; and φ depicts the initial phase of the displacement of the vibration. In Equation (5), $A_{0}$ is the initial maximum vibration amplitude; $\beta_{n}L$ = 4.73, 7.85, 11.00… (while n equals to 1,2,3…, respectively). When considering the vibration is in fundamental mode (that is 1st order mode), then we have n=1, $\beta_{1}L=4.73$. In this case, $2\pi f_{RO}=\omega_{1}\sqrt{1-\zeta^{2}}$, so the relationship between $f_{RO}$ and $f_{1}$ is $f_{RO}=f_{1}\sqrt{1-\zeta^{2}}$. Supposing φ is 0, the vibration signal at the position with x=0 can be expressed as below

(6) $$y(t)=y(x,t)|{}_{x=0}=A_{0}e^{-\frac{\zeta }{\sqrt{1-\zeta^{2}}}\cdot 2\pi f_{RO}t}\cos(2\pi f_{RO}t)$$

This is the vibrating signal that will be picked up by a fiber-coupled SMLD. The output signal from the fiber-coupled SMLD will be used to retrieve the fundamental resonant frequency $f_{RO}$ contained in y(t).

### 2.3. Capture y(t) Using Fiber-Coupled SMLD

**Figure 2:**
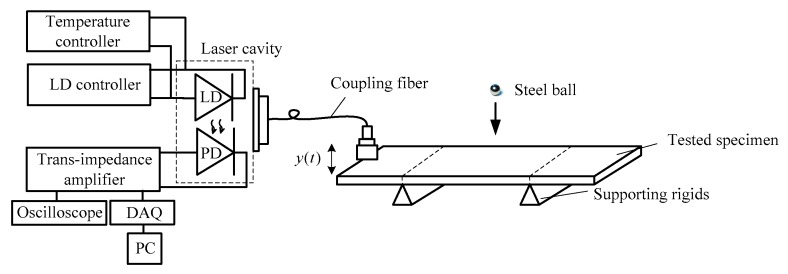
Schematic fiber-coupled SMLD.

The fiber-coupled SMLD system for capturing the vibration signal *y*(*t*) from the test specimen and obtaining $f_{RO}$ is shown in Figure 2. The system mainly consists of a LD, coupling fiber and the tested specimen. The LD is at DC biased with the LD controller. The temperature controller is used to stabilize the temperature of the LD. The emitting laser from the LD is focused onto the left end of specimen. A small portion of the light will be back-scattered or reflected by the specimen and re-enter the LD internal cavity. Both the amplitude and frequency of the LD power are modulated by the movement of the specimen. This modulated LD power (denoted by P(t)) is referred to as an SMI signal which is detected by the photodiode (PD) packaged in the rear of the LD and amplified by a trans-impedance amplifier, then recorded by an oscilloscope or collected by personal computer via analog–digital data acquisition (DAQ) card.

The widely accepted mathematical model for an SMLD is presented below [24,25,26]. The physical meanings of the parameters used in the model are presented in Table 1.

(7) $$\phi_{F}(t)=\phi_{0}(t)-C\sin\left[\phi_{F}(t)+\arctan(\alpha )\right]$$

(8) $$G(t)=\cos(\phi_{F}(t))$$

(9) $$P(t)=P_{0}[1+mG(t)]$$

where $\phi_{0}(t)$ is linked to the vibrating signal y(t) generated by the test specimen via

(10) $$\phi_{0}(t)=4\pi y(t)/\lambda_{0}$$

where $\lambda_{0}$ is the wavelength of the laser at free running.

Equations (7)–(10) describe the relationship between the signal y(t) (input to the SMLD) and P(t) (output of the SMLD). Typically, if y(t) exhibits an oscillation of frequency $f_{RO}$, P(t) will exhibit periodic waveform of the same frequency. Therefore, by applying Fast Fourier Transform (FFT) on P(t), $f_{RO}$ can be retrieved by the first peak from the amplitude spectrum of signal from P(t). In the next, we will present how to design the system so that to achieve an optimal measurement for Young’s modulus.

## 3. System Design

In order to have the vibrating signal *y*(*t*) detected effectively by the self-mixing signal, attentions must be paid to the following points during the system design. Firstly, the specimen should vibrate in the fundamental mode. Second, the maximum vibration magnitude must fall into the range required by the fiber-coupled SMLD. Furthermore, the SMLD should be insured to work in stable operation [27].

### 3.1. Mechanical Supporting for the Specimen

**Figure 3:**
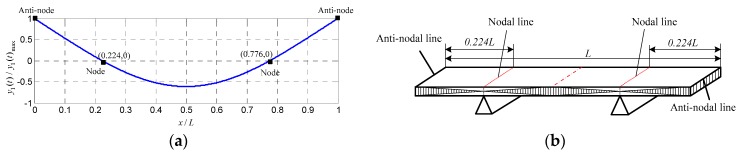
(**a**) Normalized 1st-order mode vibration of a free-free rectangular specimen; and (**b**) mechanical support for achieving 1st-order vibration.

It can be seen from Figure 3a that the two points with x/L=0.224 and x/L=0.776 are zero-cross points. They are called “nodes”. Thus, the two nodal lines indicated in Figure 3b on the specimen are chosen as the mechanical supporting position in order to have it vibrate only in 1st-order. The points with x/L=0 and x/L=1 in Figure 3a are called “anti-nodes”. One of the anti-nodes on anti-nodal line was chosen as the reference point at which the laser hits so that to pick up the vibrating signal y(t) and then generate the corresponding SMI signal P(t).

### 3.2. Steel Ball for Stimulation

A steel ball is used as the stimulator for exciting the specimen in vibration. For a given specimen, $A_{0}$ in Equation (6) is determined by the radius ($R_{steel}$) of the ball meanwhile limited by the detection range of the fiber-coupled SMLD. Hence, we need to build the relationship between $R_{steel}$ and system-associated parameters. The detection range is mainly limited by the bandwidth of PD, its associated electronics and the DAQ card. Normally, PD’s maximum detection frequency is around 10 MHz, the detection circuit currently used in our experiment has bandwidth of 4 MHz and the DAQ card we used is NI USB-6361 with 2 MHz sampling rate. We denote the overall detection bandwidth of the SMLD as $B_{D}$. Thus, the sampling frequency ($f_{s}$) in DAQ should be $f_{s}=2B_{D}$ at least. Then we consider the bandwidth of an SMI signal (denoted by $B_{S}$). $B_{S}$ can be estimated according to the feature of SMI signals [25,26,28]. Since each fringe in an SMI signal corresponds to half wavelength displacement of the external target, $A_{0}$ means the number of fringes is about $8A_{0}/\lambda_{0}$ during the first vibration period ($1/f_{RO}$) in y(t). Hence, we can roughly estimate the fringe frequency as $8A_{0}f_{RO}/\lambda_{0}$. Further considering the SMI fringe is saw-tooth-like, the harmonics of the fringe frequency can go up at least 30th-order. Thus, we can express *B_s_* roughly as:

(11) $$B_{s}=240A_{0}f_{RO}/\lambda_{0}$$

The signal bandwidth must not exceed the one of the system, that is, we should have $B_{S}\leq B_{D}$. Thus, the maximum *A*_0_ can thus be approximately determined by

(12) $$A_{0}\leq \frac{f_{s}\lambda_{0}}{480f_{RO}}$$

**Figure 4:**
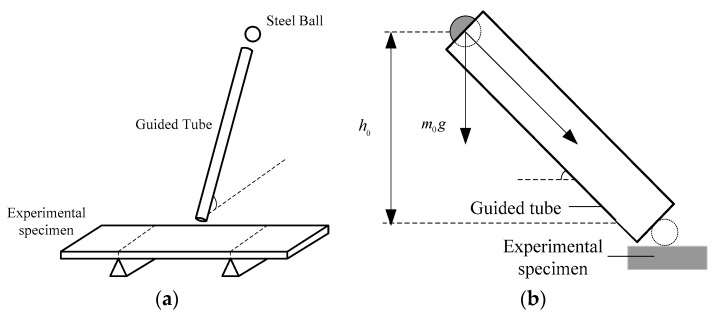
(**a**) Set-up for the generation of the excitation; and (**b**) left-view of the excitation system.

Next, we will consider the relationship between *A*_0_ and the ball’s size $R_{steel}$. In our design, the ball moves down along a guided tube and hits onto the center of the specimen. The set-up for the mechanical excitation part is shown is Figure 4. The tube is installed with a tilt angle with respect to the specimen’s plane. When the ball hits onto the specimen, an impulsive force (denoted by F) will be generated and thus cause a corresponding $A_{0}$. For the given specimen with the dimension shown in Figure 1 and $A_{0}$ determined above, *F* is expressed as below by solving the bending moment equations [29],

(13) $$F=\frac{45.432\cdot \frac{mf_{RO}{}^{2}L^{3}IA_{0}}{bh^{3}}\cdot (1.000+\frac{6.585h^{2}}{L^{2}})-2qa(a^{3}+6a^{2}L-6aL^{2}+L^{3})}{3a{\left(L-2a\right)}^{2}}$$

where I is the inertia moment of the specimen and equals to $bh^{3}/12$; q is the uniformly distributed load and equals to m/L; a=0.224L. Equation (13) tells that F is determined by $A_{0}$, $f_{RO}$ and the parameters related to the specimen.

*F* is also determined by the initial height $h_{0}$ for relieving the ball and the ball related parameters. Let us denote $m_{0}$ as the mass of the ball. According to Theorem of Momentum and Impulse and Newton’s Second Law, we have:

(14) $$(F-m_{o}g)t_{d}=m_{o}\sqrt{2gh_{0}}$$

where $m_{o}=\frac{4}{3}\pi \rho_{steel}R_{steel}{}^{3}$; $t_{d}$ is the time of collision and can be determined as 0.004 s. Thus, the radius of the steel ball can be expressed as

(15) Rsteel=3Ftd4πρsteel(gtd+2gh0)3

After combining Equations (12), (13) and (15), a suitable $R_{steel}$ can be worked out. A ball with this size can generate a y(t) with $A_{0}$ meeting the detection requirement of an SMLD.

### 3.3. Requirements for SMLD

**Figure 5:**
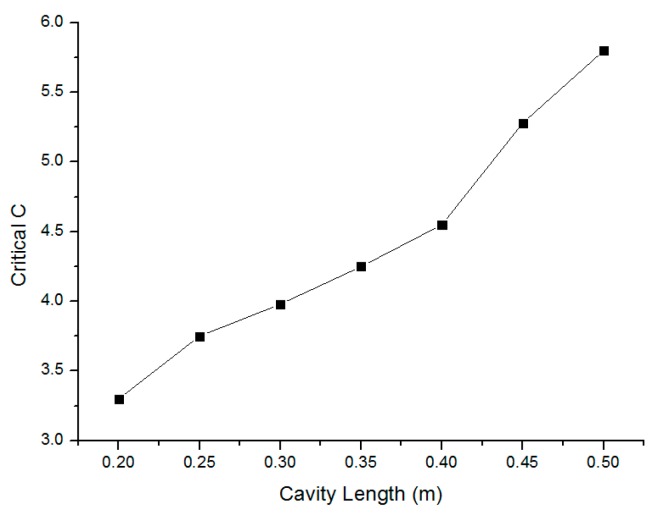
Stability boundary described by *C* and *h*_0_ when the injection current is 52.5 mA.

The stability of an SMLD is studied in work [27]. It shows that the stability boundary is determined by the injection current, feedback level and the external cavity length. An SMLD is stable only when it operates below the stability boundary. In our system, the LD is L785P090 (785 nm, 90 mW) with injection current 52.5 mA, which is 1.5 times the threshold value (35 mA). We measured the system stability boundary using the experimental method presented in [27] by varying the system feedback level and the external cavity length, as shown in Figure 5. We choose the cavity length as 0.5 m so that the SMLD can be stable over a wide feedback level.

Note that it is better to use an attenuator to adjust the feedback level C to be around 3, in this case SMI signals can be clear without relaxation oscillation.

In summary, the following three steps are important for designing a suitable fiber-coupled SMLD system for Young’s modulus measurement.

- Step 1: Measure the stability boundary of the SMLD system and from which to determine a suitable external cavity length to place the tested specimen.
- Step 2: Estimate the maximum magnitude $A_{0}$ by Equation (12). Note that a low $f_{RO}$, e.g., can be used for the estimation.
- Step 3: Calculate the size of the steel ball $R_{steel}$ using Equations (13) and (15) and $A_{0}$.

## 4. Simulations

In order to verify the concept presented above, we firstly perform simulations with the aim to show the feasibility for measuring Young’s modulus by the fiber-coupled SMLD.

The specimen we used is a rectangular brass bar (with L = 138.35 mm, b = 12.06 mm, h = 2.23 mm, m = 30.65 g) and its Young’s modulus is estimated as 120 Gpa from the literature [30]. Thus, its $f_{RO}$ is calculated as 444 Hz by Equation (1).

For simulations, the parameters associated to the SMLD are set as $f_{s}$ = 3 MHz (considering the bandwidth of the detection circuit used for experiments is 3 MHz), $\lambda_{0}$ = 785 nm, and we choose C = 3, α = 3, and the external cavity length is $h_{0}$ = 0.5 m.

Based on above design procedure, we have maximum $A_{0}$ = 11.05 um using Equation (12). According to Equation (6), if we let ζ = 0.0015, y(t) generated by the brass specimen is expressed as

(16) $$y(t)=11.05\cdot e^{-4.6t}\cos\left(2\pi \cdot 444\cdot t\right)$$

From y(t), we can obtain $\phi_{0}(t)$ through Equation (10), then $\phi_{F}(t)$ by Equation (7), and finally, we can get G(t) using Equation (8) Note that in the simulation, we use G(t) to replace P(t). In practice, G(t) can be gained by normalizing P(t) through Equation (9).

**Figure 6:**
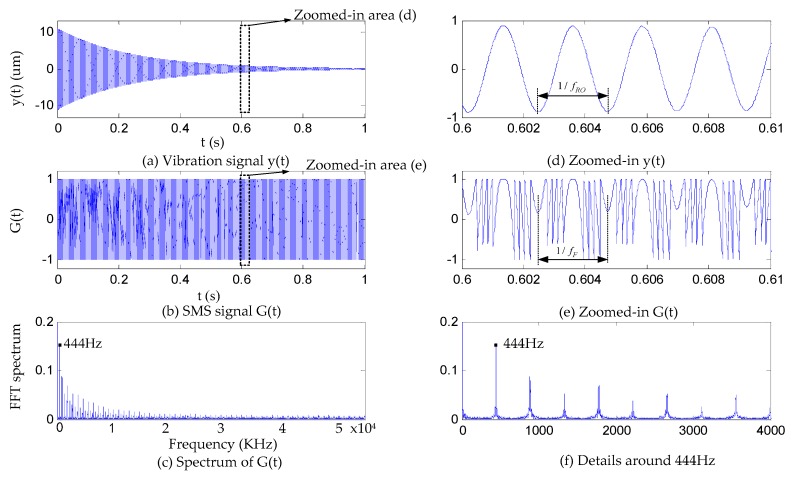
(**a**–**f**) Simulation results.

Since the FFT frequency resolution (denoted by $R_{data}$), the sampling data length for FFT (denoted by $L_{data}$) and $f_{s}$ have a relationship; that is, $R_{data}=\frac{f_{s}}{L_{data}}$. To measure $f_{RO}$ = 444 Hz, the frequency resolution should be at least 1 Hz, so $L_{data}$ should be equal to 3,000,000 at the same time. We firstly generated y(t) by Equation (16) with 5 million specimens as shown by Figure 6a. The corresponding SMI signal G(t) was simulated using Equations (7), (8) and (10) and plotted in Figure 6b. We applied FFT on G(t) and gained its amplitude spectrum shown in Figure 6c. Figure 6d,e shows the zoomed-in area indicated in Figure 6a,b,f, which shows the details of the spectrum around 444 Hz.

From the time domain in Figure 6b, it can be observed that the period (noted by $1/f_{RO}$ in Figure 6d) of damping vibration y(t) equals to the fundamental period (noted by $1/f_{F}$ Figure 6e) of SMI signal G(t). The fundamental frequency can be easily found from the spectrum of G(t) by detecting the first peak.

**Figure 7:**
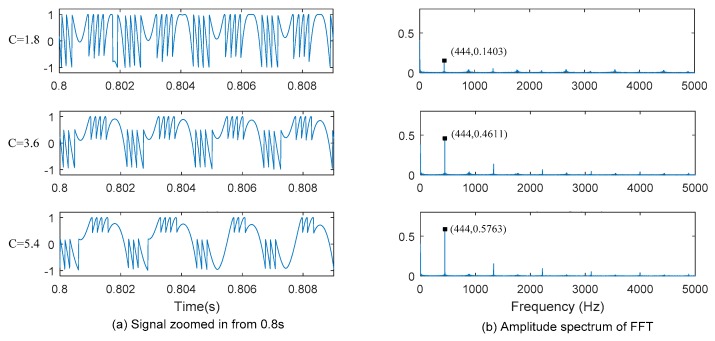
FFT spectrum of G(t) under different feedback level. (**a**) Signal zoomed in from 0.8 s (**b**) Amplitude spectrum of FFT.

We also performed the simulations by considering the SMLD under different feedback levels C, which are 1.8, 3.6 and 5.4. Part of signal from 0.8 s for four periods is shown in Figure 7a. Other parameters for simulations are same as the ones used in Figure 6. The spectrums of corresponding G(t) under different feedback levels are shown in Figure 7b.

From Figure 7, decrease in the amplitude of the dominant fundamental frequency component was found in each feedback level, but it is still very clear as long as C was chosen larger than 1, *i.e.*, the moderate or strong feedback regime. However, it is found that when the system is working at weak feedback level, the fundamental frequency component cannot be separate from other frequency components. Thus, the system must be kept working in a moderate or relatively strong feedback level, but within the range where system can stably work, *i.e.*, around 5.8, according to the requirement of SMLD in Figure 5. In practice, it is very rare in the experiments C exists smaller than 1 until an attenuator was used. Thus, the fundamental resonant frequency in input signal can finally smoothly be retrieved from the output of the SMLD measuring system through FFT.

## 5. Experiments

### 5.1. Experimental Set-up and Results

**Figure 8:**
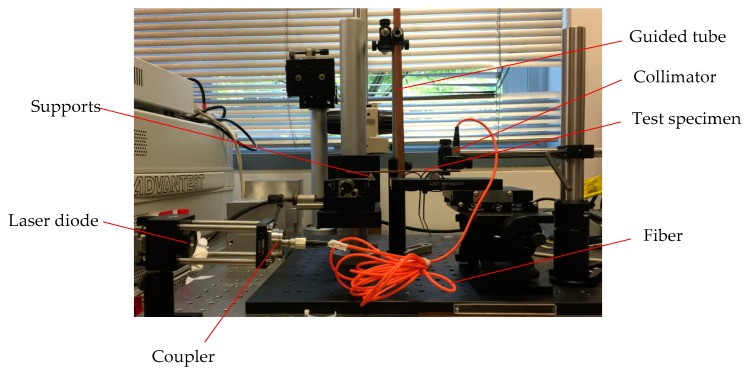
Experimental set-up of fiber-coupled SMLD.

The overall experimental set-up is shown in Figure 8. The experiments were conducted on two different material specimens, one of which is a rectangular brass bar with L = 138.35 mm, b = 12.06 mm, h = 2.23 mm and m = 30.65 g and the other one is an aluminum alloy 6061 specimen with L = 132.43 mm, b = 12.24 mm, h = 2.00 mm and m = 8.70 g. The radius of the steel ball for experiments was set as $R_{steel}$ = 3 mm within the maximum limit calculated by using Equations (12), (13) and (15). Then experiments can be performed using the following steps.

- Step 1: Install the LD onto a laser mount; set the bias current on the laser controller (LTC100-B from THORLABS) as 52.5 mA and the temperature on the temperature controller (TED200C from THORLABS) is stabilized to 25 ± 0.1 °C.
- Step 2: Install a specimen to be tested and use a coupler (PAF-X-2-B from THORLABS) connected with a step-index multimode fiber optic patch cable (M67L02 from THORLABS) with an adjustable aspheric FC collimators (CFC-2X-B from THORLABS) at the other end to adjust the distance between the specimen and the LD to form an external cavity with 0.5 m long.
- Step 3: Adjust the LD mount so that the fiber-coupled SMLD can be operated in a moderate feedback level by observing the waveform of the SMI signal.
- Step 4: Place the steel ball on the up end of the guided tube and release it. As a result, the specimen is stimulated into vibration. Correspondingly, an SMI signal is produced by the SMLD and recorded by the oscilloscope and the computer through the DAQ card. A LabVIEW script programmed for sampling the SMI signal is set to wait for collecting the signal.

**Figure 9:**
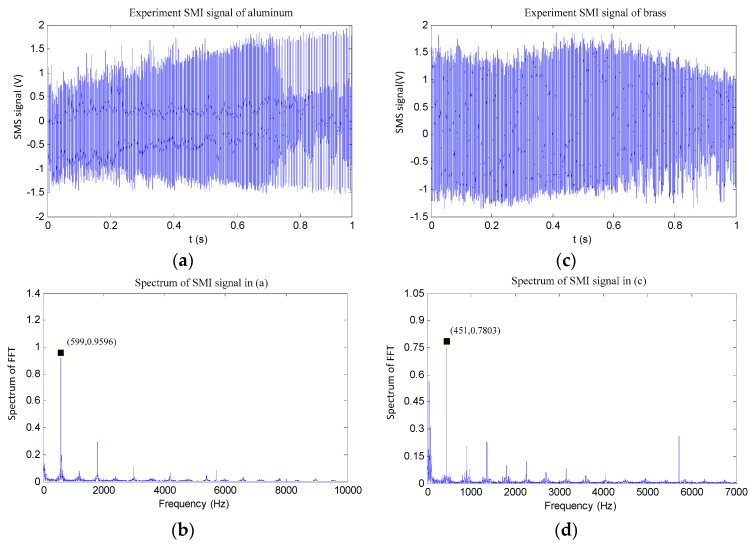
(**a**,**b**) Experimental results of aluminum 6061; and (**c**,**d**) experimental results of brass.

For each specimen, Step 4 was repeated 10 times. Thus, 10 pieces of SMI signals were collected and the corresponding spectrums are calculated by applying FFT. For illustration, we show one pair of the experimental results for each specimen in Figure 9a–d. The sampling rates were all set as 200 KHz during the experiments. The data length for each piece of signal is 200,000 points. Hence, the resolution of each spectrum can reach to 1 Hz. From the spectrums in Figure 9d, the first peak is detected as the fundamental resonant frequency $f_{RO}$. It is characterized as the highest peak in the spectrum. The Measurement details of fundamental resonant frequency $f_{RO}$ for the two spectrums (aluminum 6061 and the brass) are shown in Table 2.

For the aluminum 6061 specimen, the measured resonant frequency values vary from 597 Hz to 599 Hz and it is from 450 Hz to 452 Hz for the brass. It can be seen that the proposed method can achieve the measurement for $f_{RO}$ with high repeatability. We then use the obtained $f_{RO}$ and Equations (1) and (2) to calculate the Young’s modulus and the results are also presented in Table 2. We use standard deviation to describe the measurement accuracy, which is calculated by

(17) $$\sigma =\sqrt{\frac{1}{N}\sum_{i=1}^{N}{\left(x_{i}-\mu \right)}^{2}}$$

where $x_{i}$ refers to each measurement result of$f_{RO}$, or the calculated E shown in Table 2. *N* = 10. μ is the mean value over the measured 10 values. From the standard deviation given in Table 2, the measurement accuracy for $f_{RO}$ and E are respectively calculated by (σ/μ) % as 0.23% and 0.25%. The Young’s moduli are 70.0 GPa and 116.7 GPa, respectively, for aluminum 6061 and the brass specimen. The two results fall in the ranges of 69–72 GPa and 102–125 GPa reported in the literature [31].

### 5.2. Comparison with Tensile Testing

Six standard dog-bone shaped flat specimens with gauge length 25 mm, width 10 mm and thickness 2 mm were taken from the above mentioned aluminum 6061 and brass respectively [32]. Tensile tests were performed on an Instron 5566 testing machine at room temperature with an initial strain rate of 10^−3^/s. The load values were recorded by the load cell of the Instron machine. To ensure the measurement accuracy of Young’s modulus, DANTEC digital image correlation (DIC) system was adopted to record the displacement of tensile specimens during the tests. Before testing, random speckle patterns were generated on the specimen surfaces by spray painting. The overall displacement of the entire gauge regions of the specimens was recorded by two high speed cameras facing the speckled surfaces at a frame rate of 5 Hz. The images were 2448 by 2448 pixels with an 8-bit dynamic range. ISTRA 4D software was used to analyze the images and obtain extension values of the gauge regions. The load obtained from the Instron machine and the extension obtained from the DIC system were used to calculate stress and strain values. The stress–strain curves were plotted afterwards. Young’s modulus was obtained from the elastic deformation region of the stress–strain curves.

**Figure 10:**
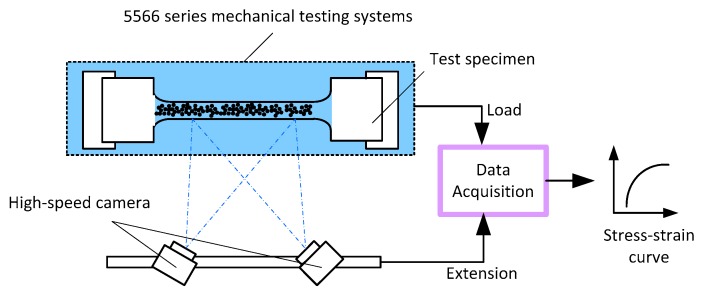
Schematic experimental set-up for tensile testing.

**Figure 11:**
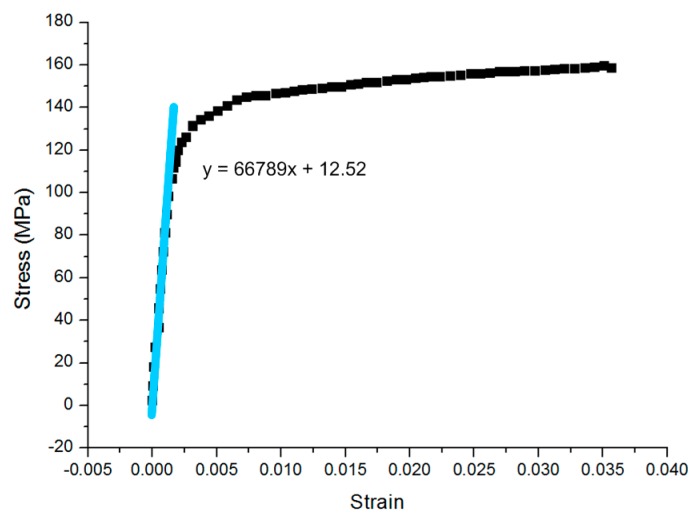
Stress–strain curve for an aluminum 6061 specimen obtained from tensile testing.

Figure 10 is the schematic experimental setup for tensile testing. As an example, Figure 11 shows one of the stress–strain curves obtained for aluminum 6061. The Young’s modulus can be read by the slope of the linear region on the curve. It can be seen that the linear region can be fitted by a linear equation y = 66789x + 12.52, whose slope is around 66.79 GPa, which is the Young’s modulus value.

The results of the measured Young’s modulus are presented in Table 3.

By comparing the results in Table 2 and Table 3, it can be seen that the Young’s modulus obtained by the fiber-coupled SMLD for the two different materials are quite close to the results measured by the traditional method—tensile testing. However, relative large deviations are found from the tensile testing with 6.2 GPa for aluminum 6061 and 7.9 GPa for brass, and the corresponding accuracy are 9.2% and 6.5%, while the proposed fiber-coupled SMLD system is able to measure the Young’s modulus with a satisfied accuracy, 0.23% for aluminum 6061 and 0.25% for the brass. In addition, the SMLD system needs only one specimen for each material to obtain the Young’s modulus but multiple specimens are required by tensile testing for higher accuracy.

## 6. Conclusions

An optical method based on SMLD is developed for Young’s modulus measurement. Detail design procedures are presented. Both simulation and experiments show that the proposed measurement method can achieve Young’s modulus with accurate results. The Young’s modulus for material aluminum 6061 and brass are measured using the proposed fiber-coupled SMLD as 70.0 GPa and 116.7 GPa, showing a good agreement with the standards reported in the literature and yielding a much smaller deviation (0.16 GPa and 0.29 GPa) and a higher accuracy (0.23% and 0.25%) in contrast to the traditional tensile testing. In addition, unlike tensile method, the proposed approach only acquires one sample for experiments, and can be performed in a non-destructive way. The proposed fiber-coupled SMLD system for Young’s modulus measurement is characterized as compact structure, fast measurement and non-contact technique. By cooperating advanced signal processing and fast DAQ card, this method can achieve very high measurement accuracy. With the fiber-coupled SMLD, the system is quite easy to be installed and can be used in many application cases.

## Figures and Tables

**Table 1:** Physical meanings of Parameters.

Parameters	Physical Meaning	Unit
t	Time index.	s
$\phi_{F}(t)$	Laser phase with feedback	rad
$\phi_{0}(t)$	Feedback level factor	rad
C	Line-width enhancement factor	-
α	Interference function which indicates the influence of the optical feedback	-
G(t)	Interference function which indicates the influence of the optical feedback	-
m	Modulation index for the laser intensity (typically m≈0.001)	-
$P_{0}$	Laser intensity emitted by the free running LD	-
P(t)	Laser intensity when LD with optical feedback	-

**Table 2:** Measurement results.

	Specimen	Aluminum 6061		Brass	
Times (N)		$f_{RO}$ (Hz)	E (GPa)	$f_{RO}$ (Hz)	E (GPa)
1		599	70.2	451	116.6
2		598	70.0	450	116.1
3		599	70.2	451	116.6
4		598	70.0	451	116.6
5		597	69.7	452	117.1
6		598	70.0	451	116.6
7		599	70.2	451	116.6
8		598	70.0	452	117.1
9		599	70.2	451	116.6
10		598	70.0	451	116.6
Mean (μ)		598	70.0	451	116.7
Standard deviation (σ)		0.68	0.16	0.57	0.29

**Table 3:** Results of Young’s modulus (GPa) by tensile testing.

	Times (N)	1	2	3	4	5	6	Mean (μ)	Standard Deviation (σ)	Accuracy (σ/ μ%)
Specimen										
Aluminum 6061		60.6	64.4	76.2	67.0	73.9	63.0	67.6	6.2	9.2
Brass		120.3	125.6	133.4	118.6	109.6	119.4	121.1	7.9	6.5

## Abbreviations

The following abbreviations are used in this manuscript:

SMLD	Self-Mixing laser diode
LD	Laser Diode
PD	Photodiode
SMI	Self-mixing interferometry
FFT	Fast Fourier Transform
DAQ	Data Acquisition
